# Comparative analysis of inverted repeats of polypod fern (Polypodiales) plastomes reveals two hypervariable regions

**DOI:** 10.1186/s12870-017-1195-z

**Published:** 2017-12-28

**Authors:** Maria D. Logacheva, Anastasiya A. Krinitsina, Maxim S. Belenikin, Kamil Khafizov, Evgenii A. Konorov, Sergey V. Kuptsov, Anna S. Speranskaya

**Affiliations:** 1 M.V. Lomonosov Moscow State University, 119991 Moscow, Russia; 2 Moscow Institute of Physics and Technology, Dolgoprudny, 141700 Moscow Region Russia; 3 Federal Budget Institution of Science Central Research Institute of Epidemiology of The Federal Service on Customers, 111123 Moscow, Russia; 4 Vavilov Institute of General Genetics, Russian Academy of Sciences, 119991 Moscow, Russia

## Abstract

**Background:**

Ferns are large and underexplored group of vascular plants (~ 11 thousands species). The genomic data available by now include low coverage nuclear genomes sequences and partial sequences of mitochondrial genomes for six species and several plastid genomes.

**Results:**

We characterized plastid genomes of three species of *Dryopteris*, which is one of the largest fern genera, using sequencing of chloroplast DNA enriched samples and performed comparative analysis with available plastomes of Polypodiales, the most species-rich group of ferns. We also sequenced the plastome of *Adianthum hispidulum* (Pteridaceae)*.* Unexpectedly, we found high variability in the IR region, including duplication of *rrn16* in *D. blanfordii*, complete loss of *trnI*-GAU in *D. filix-mas*, its pseudogenization due to the loss of an exon in *D. blanfordii.* Analysis of previously reported plastomes of Polypodiales demonstrated that *Woodwardia unigemmata* and *Lepisorus clathratus* have unusual insertions in the IR region. The sequence of these inserted regions has high similarity to several LSC fragments of ferns outside of Polypodiales and to spacer between *tRNA-CGA* and *tRNA-TTT* genes of mitochondrial genome of *Asplenium nidus*. We suggest that this reflects the ancient DNA transfer from mitochondrial to plastid genome occurred in a common ancestor of ferns. We determined the marked conservation of gene content and relative evolution rate of genes and intergenic spacers in the IRs of Polypodiales. Faster evolution of the four intergenic regions had been demonstrated (*trnA- or*f42, *rrn16*-*rps12, rps7*-*psbA* and *ycf2*-*trnN*).

**Conclusions:**

IRs of Polypodiales plastomes are dynamic, driven by such events as gene loss, duplication and putative lateral transfer from mitochondria.

**Electronic supplementary material:**

The online version of this article (doi: 10.1186/s12870-017-1195-z) contains supplementary material, which is available to authorized users.

## Background

Сhloroplast genomes (plastomes) of land plants are generally conserved in their size, gene content and order. The evolutionary origin of chloroplasts, as well as mitochondria, traces back to ancient endosymbiotic bacteria that consequently greatly reduced the genome, and contain only a small proportion of the ancestor’s genes [1, 2].

Plant plastomеs possess a quadripartite structure composed of large single-copy (LSC) and small single-copy (SSC) regions divided by two parts of inverted repeat (IR) [2, 3]. Plastome size of higher plants is usually around 150,000 bp in length and comprise approximately 120–130 genes, among which about 75 genes encode proteins of photosystem I and II, as well as for other proteins, involved in photosynthesis [see, for example, [4]], while other genes encode ribosomal RNA and proteins and transfer RNA. The highest deviations of the gene order and content in plastomes of land plants have been reported in non-photosynthetic species, in which extremely reduced plastomes were found - up to 11 Kbp [5]. However, in photosynthetic plants lineage-specific gene losses, as well as translocations that change the gene order were also observed [6–8].

IRs are usually regarded as the most stable part of the plastome. Indeed, the substitution rate of the IR sequences is the lowest compared to single copy regions [3]; the plastomes where IRs are absent (e.g. IRLC clade of Fabaceae) exhibit elevated substitution rates [9] and vice versa, genes that were translocated from SC to IR slow down their substitution rate [10]. IRs typically range in size from 15 to 30 kbp and contain a core set of genes consisting of four rRNA genes (*4.5S*, *5S*, *16S* and *23S rRNA*), tRNA genes (*trnAUG*, *trnI-GAU*, *trnN-GUU*, *trnR-ACG* and *trnV-GAC*). The IRs of many land plants also contain a number of other genes as a result of lineage-specific expansions and contractions [3]. Few evolutionary lineages demonstrate large-scale expansions (exceeding several kbp and containing numerous genes) of the IR [3, 11]. In particular, several overlapping inversions affect size, gene content and order of the IR in leptosporangiate ferns, the clade that includes most fern species [8, 10].

Another evolutionary event that can affect plastome structure is horizontal gene transfer (HGT). HGT between nucleus, mitochondria and plastids has been shown to occur with a high rate and contributed significantly to the plant genome evolution by relocating and refashioning of the genes and consequently contributing to genetic diversity. Transfers of DNA fragments from the mitochondria or plastids to the nucleus are the most common reported ones [12–16]. The mitochondrial genomes are also often invaded by plastome-derived sequences; the presence of DNA from nuclear genomes also has been shown in a number of lineages of flowering plants [17–24] and ferns [25]. Translocations of mtDNA fragments to plastid genome are much rarer. Currently, only two cases, all from flowering plants, are known. In *Daucus carota* (order *Apiales*, Apiaceae) plastome a ~1.5 Kb region with high similarity to *Vitis vinifera* (order *Vitales*) mitochondrial sequence was found [26]. This region did not contain any typical plastid genes. Characterization of *Daucus* mitogenome and screening of plastomes of other *Apiaceae* suggest that it was inserted from the mitochondrial genome to the plastome in a common ancestor of the genus *Daucus* [27]. Another example is the horizontal gene transfer is a 2.4-kb segment of mitochondrial DNA into the *rps2–rpoC2* intergenic spacer of the plastome of *Asclepias syriaca* (*Apocynaceae*) [28]. Thus, unlike the mitochondrial genomes, which are affected by insertions of plastid and nuclear sequences, the plastomes of flowering plants are infrequently profited by DNA transfer from the other cell compartments [22, 29, 30].

Extant ferns are non-seed vascular plants for which 45 families are currently known (with approx. 280 genera), of which more than half belong to the order Polypodiales, in line with the classification of [31]. The majority of Polypodiales species fall into two large sister clades - Eupolypods I and Eupolypods II, and the remaining to families *Pteridaceae*, *Dennstaedtiaceae*, *Saccolomataceae*, *Lindsaeaceae* (basal clade) [32]. Wolf et al. [8] for the first time examined structure of the plastome across few widely ranged representative fern taxa but no one has represented the Polypodiales. Zhu et al. [3] analyzed the evolutionary rate and shifts in IR boundaries of land plants, including seven ferns but also no Polypodiales were considered. Raman et al. [33] characterized *Cyrtomium falcatum* plastid genome and found some differences with congeneric species *C. devexiscapulae* in tRNA gene content and start codons. For 24 fern samples, five of which were Polypodiales species (other represented ten extant orders), the part of LSC region (*rpoB-psbZ*) was analyzed and considerable genomic changes for distant species (belonging to different orders) were found [34]. A comparison of a few taxon-wide fern plastomes (of Lycopodiophyta, Psilotopsida, Equisetopsida, Marattiopsida and Polypodiopsida) showed that some lineages have experienced multiple IR changes including expansions and inversions while others demonstrated the stasis [35]. Recently many new sequences of fern plastomes (including Polypodiales species) were released. However, the corresponding study reports only the results of phylogenetic analysis of these sequences, without detailed analysis of their gene content and structure [36].

This clearly indicates that the diversity of plastome structures in ferns is insufficiently explored. With this premise, we characterized four additional plastome sequences from Polypodiales, three from *Dryopteris* and one from *Adianthum*, and performed comparative analysis of all available fern plastomes.

## Results

**Fig. 1:**
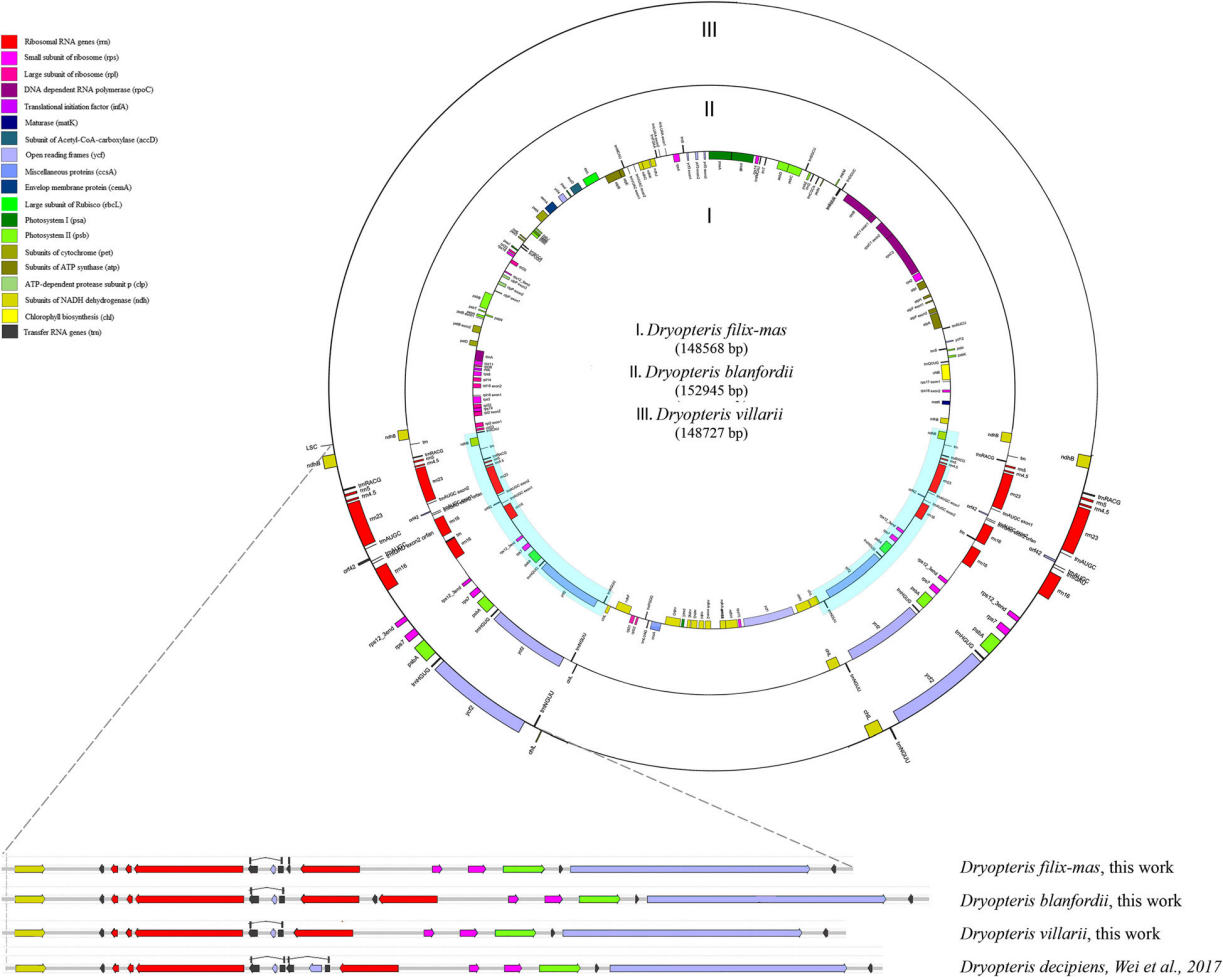
Gene maps of the Dryopteris plastomes. Genes drawn inside the circle are transcribed clockwise, and those outside the circle are transcribed counter clockwise

We sequenced and assembled de novo new plastome sequences for three Eupolypods I species: *Dryopteris filix-mas*, *Dryopteris blanfordii* and *Dryopteris villarii*. The plastomes have typical quadripartite structure, are similar in their size (148,568*,* 152,945 and 148,727 bp, respectively). A total of 130 genes were annotated by DOGMA for *D. filix-mas*, including 91 protein-coding genes (5 of them are duplicated in IRs), 25 tRNA genes (5 of them are duplicated in IRs) and 4–5 rRNA genes (all of them are duplicated in IRs). Plastomes of *Dryopteris* species*,* including *D. blanfordii, D. villarii, D. filix-mas* and previously reported *D. decipiens* were identical to each other in gene content of LSC and SSC, but differed in IRs (Fig. 1).

**Fig. 2:**
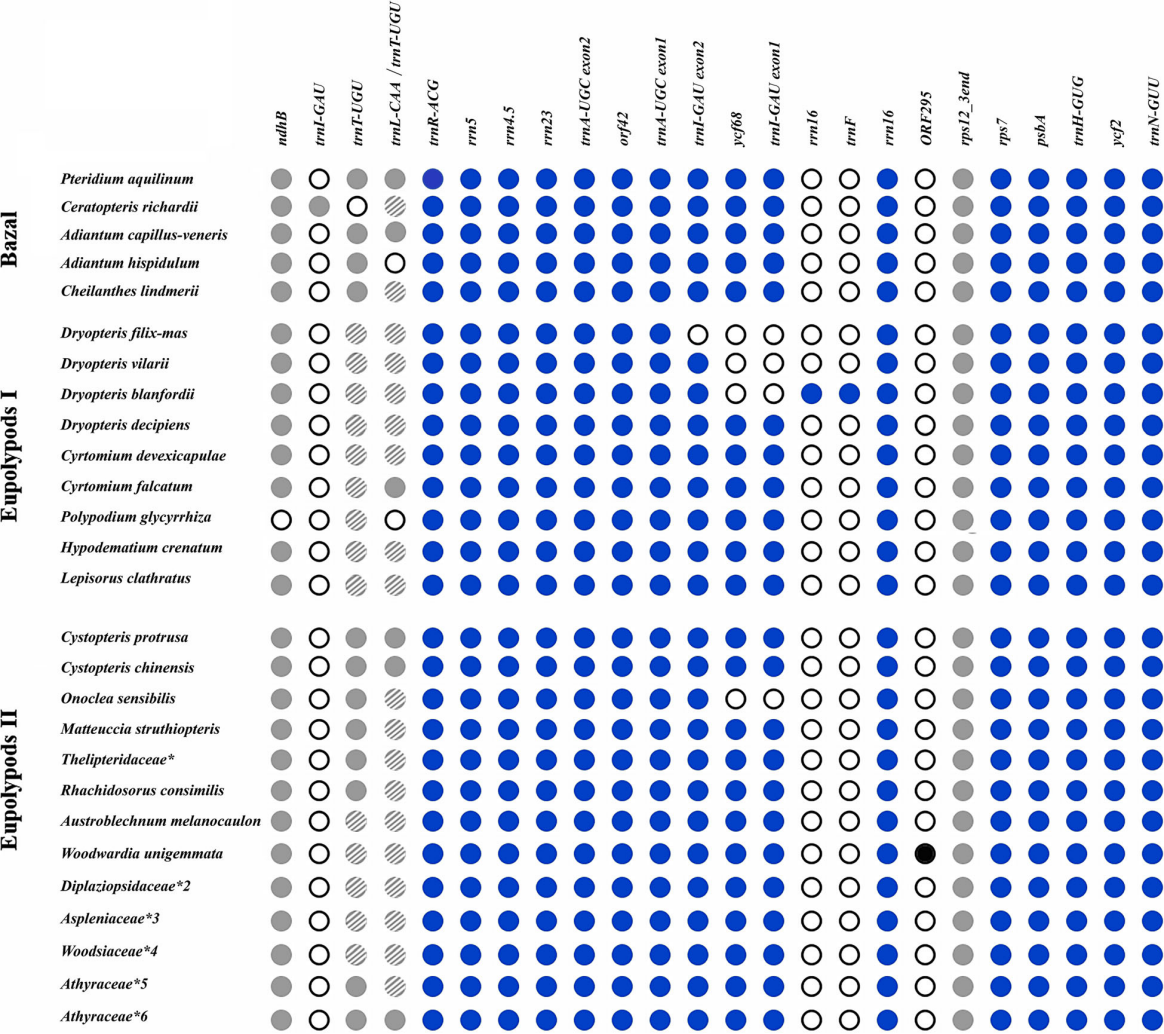
The IR gene content among Polypodiales ferns. The blue filled and open circles indicate the genes present and absent, respectively, in the corresponding species. The light-grey filled circles denote sequences that were marked as pseudogenes by DOGMA and shaded light-grey – pseudogenes that were not found by DOGMA, only by manual check using BLAST. * – *Macrothelypteris torresiana*, *Thelypteris aurita*, *Stegnogramma sagittifolia*, *Cyclosorus procerus*, *Ampelopteris prolifera*; *2 – *Homalosorus pycnocarpos*, *Diplaziopsis javanica*, *Diplaziopsis cavaleria*; *3 – *Asplenium prolongatum*, *Asplenium pekinense*, *4 – *Woodsia macrochlaena*, *Woodsia polystichoides*; *5 – *Conopteris opaca* (syn. *Athyrium opacum*) and all Deparia species of Athyraceae, *6 – *Anisocampium sheareri* (syn. *Athyrium sheareri*) and all other *Athyrium* species of Athyraceae

We also sequenced one the plastome of *Adianthum hispidulum*, from Pteridaceae, the basal group relative to eupolypods*.* The complete plastome sequence of *A. hispidulum* was 151,327 bp in length, consisted of an LSC (83,188 bp), SSC (21,459 bp), and IRs (23,340 bp). The gene content in the plastome of *A. hispidulum* slightly differs from that of congeneric species *A. capillus-veneris* (published in [37]) – in *A. hispidulum trnT-UGU* gene (located in IR) is completely absent while in *A. capillus-veneris* it is represented by a pseudogene (Fig. 2).

Comparative dataset of IR sequences which includes all plastomes for Polypodiales available in the public sequences databases and our new data comprises 45 sequences: 31 belong to Eupolypods I, 9 – to Eupolypods II and the remaining 5 - to Pteridaceae or Dennstaedtiaceae. We have made re-annotation of published Polypodiales plastomes. The IR/SSC border of all species lies within the *ndhF* gene and IR/LSC - within *ndhB* gene. The similar IR borders were previously defined for many groups of monilophytes (Psilotales, Ophioglossales, Equisetales, Marattiales and Polypodiopsida) [35]. Commonly, chloroplast genomes of Polypodiales carry sequences (~ 246 bp) with high similarity to *ycf68* ORF in the IR regions, though they were not previously annotated by the authors. The *ycf68* is a putatively functional gene located in the *trnI-GAU* intron, which is present in many land plant chloroplast genomes but often is not annotated as its function is still unknown [38, 39]. Another putative gene, *ORF42*, was annotated in the *trnI-GAU* intron for the all species included in the analysis. *ORF42* was found previously in the intron region of *trnA-UGC* plastid gene of some flowering plant species, for example, *Veratrum patulum* O. Loes. (Melanthiaceae) [40] and *Pelargonium* × *hortorum* L. H. Bailey (Geraniaceae) [11]. The sequence with high similarity to *ORF42* was found in the mitochondrial genome of *Phaseolus*; presumably as a result of plastid-to-mitochondrion lateral gene transfer [19].

The IR structures of Polypodiales are shown in Fig. 2 and Additional file 1. The number of genes normally varies from 14 to 16. The plastomes of Polypodiales mainly accumulated the gene number variability within IR in two areas: from 0 to ~3 Kbp and from ~7 to ~11,5 Kbp regions of IR. These regions also demonstrate lower sequence similarity. The other two regions - from ~3 to ~7 Kbp and from ~11,5 Kbp till the end of IR - were largely conservative in gene number and sequence.

The 0 to ~3 Kbp region contains tRNA (pseudo)gene (Fig. 2). Though it is annotated as functional two-exon *trnT-UGU* gene in many species (e.g. 33, 37), the tRNA structure prediction with tRNAscan-SE does not support its functionality. In other species it is annotated as pseudogene (*trnT-UGU* or *trnL-CAA*) or completely missing. Manual check however shows that pseudogene is present in all species analyzed (Fig. 2) except for *Adianthum hispidulum* where it was completely lost.

In the ~7 to ~11,5 Kbp variable region three genes show partial or full deletions or duplications (*trnI-GAU*, *ycf68, rrn16*) (Fig. 2) (Additional file 1). Some species of Polypodiales have partially or completely lost *trnI-GAU* and *ycf68* (located in intron of *trnI-GAU*) namely: *O. sensibilis* (Onocleaceae) and three of four *Dryopteris* species (*D. blanfordii, D. villarii, D. filix-mas,* Dryopteridaceae). In *D. filix-mas trnI-GAU* and *ycf68* are completely lost. *D. blanfordii* has partially lost the *trnI-GAU* gene (the intron and one of exons were deleted), but has a duplication of the large part of *rrn16* gene. *D. villarii* has lost intron and one exon of *trnI-GAU* but no *rrn16* duplication. As result of deletions/duplications the IR size of *Dryopteris* species varied: IR of *D. filix-mas* and *D. villarii* were ~1450–1570 bp shorter but *D. blanfordii*, on the contrary, had IRs 647 bp longer (*Cyrtomium* species were used as reference). Surprisingly *D. decipiens,* reported in [36], has no deletions in this region*. O. sensibilis* (Onocleaceae) plastome has a deletion of *ycf68*-*trnI-GAU* region. *Onoclea* and *Dryopteris* species belong to different clades: Eupolypod II and Eupolypod I. Therefore, the losses of *ycf68*-*trnI-GAU* regions are independent events.

**Fig. 3:**
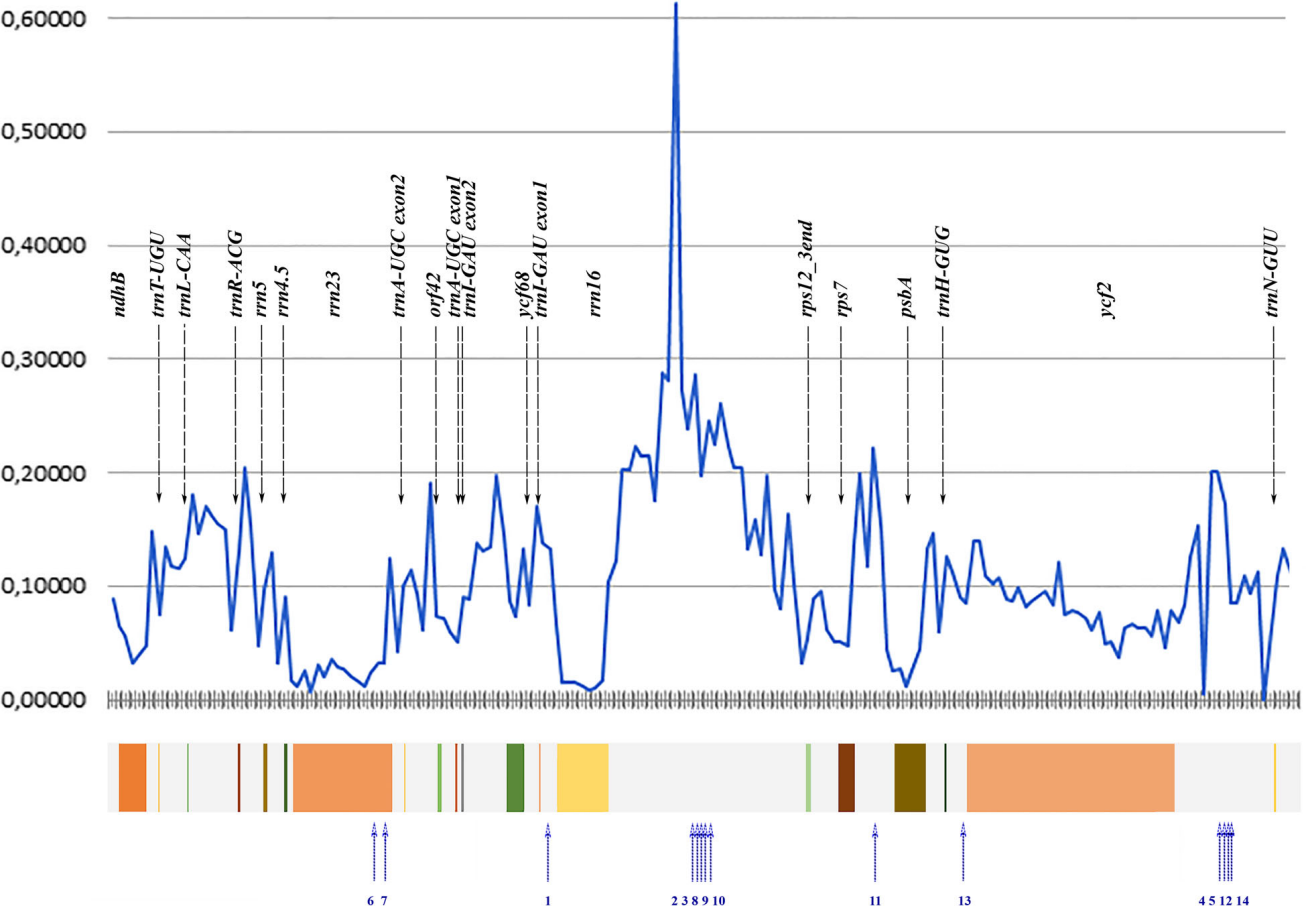
Nucleotide diversity and position of insertions among IRs of the Polypodiales. Horizontal axis indicates the coordinates within the aligned IRs of all Polypodiales. Vertical axis indicate Sliding window analysis (window = 200 b.p.) of p-distances, i.e. the proportion of nucleotide differences per site between sequences calculated by perl script made by Masafumi Nozawa. Colored boxes below marked the coding regions of genes (in the IR of averaged Polypodiales). Blue numbered arrows indicate position of insertions in IR sequences of different fern species (see also Table 2)

Due to the deletions and duplications IR size in Polypodiales varies from 22 Kbp (*Ceratopteris richardii* [KM052729]) to 26,9 Kbp (*Cystopteris protrusa,* [KP136830]. In particular, we found the most of the large insertions (370 bp and more) in the highly variable intergenic spacers mentioned above: intergene 14 (between *rrn16* and *rps12*), intergene 16 (between *rps7* and *psbA*) and intergene 19 (between *ycf2* and *trnN-GUU*), see Fig. 3. For the insertion’s length, localization and similarity to known high plant sequences see Table 1.

**Table 1:** Relative evolution rate *(ERaBLE output was normalized by rate of evolution in concatenated RNA- and protein-coding sequences)* in different regions of IR

Region	All Polypodiales	Eupolypods 1	Eupolypods 2	Dennstaedtiaceae + Pteridium
Intergene1	1,27	1,43	1,22	1,65
ndhB	2,46	3,50	2,78	2,83
Intergene2	11,52	12,55	10,86	14,59
trnT-UGU	16,59	26,18	3,11	31,63
Intergene3	2,68	3,32	2,20	3,45
trnL-CAA	2,20	3,24	2,44	2,53
Intergene3p	*5,43*	*8,53*	*6,47*	*5,13*
trnR-ACG	85,25	0,00	138,59	0,00
Intergene4	10,11	13,42	12,65	10,99
rrn5	0,15	0,22	0,27	0,11
Intergene5	7,25	0,46	8,02	7,12
Intergene6	2,35	2,49	3,46	0,93
rrn23	0,78	9,81	1,77	0,30
Intergene7	2,97	2,51	4,30	2,26
trnA-UGC	0,00	0,00	0,00	0,00
**Intergene8**	**22,93**	**10,48**	**59,98**	**11,34**
orf42	530,31	1221,25	3,38	3,77
Intergene9	0,74	1,05	0,86	0,73
trnA-UGC2	0,02	0,04	0,00	0,00
Intergene10	2,67	16,92	1,71	1,70
trnI-GAU	0,00	0,00	0,00	0,00
Intergene11	2,07	3,40	2,61	2,22
ycf68	2,87	2,55	8,39	1,07
Intergene12	68,77	41,48	245,28	15,63
trnI-GAU2	2,31	5,57	0,00	0,00
Intergene13	3,01	4,05	3,76	3,10
rrn16	0,42	0,18	0,97	0,20
**Intergene14**	**9,37**	**27,47**	**11,23**	**8,26**
rps12	1,88	2,13	2,77	1,44
Intergene15	1,43	1,48	1,24	1,59
rps7	2,20	2,32	2,65	1,83
Intergene16	**7,31**	**7,69**	**7,79**	**7,57**
psbA	1,42	1,89	1,78	1,30
Intergene17	2,57	4,21	2,63	2,91
trnH-GUG	0,35	0,86	0,00	0,01
Intergene18	3,14	1,92	3,36	4,20
**Intergene19**	**5,47**	**5,07**	**4,97**	**6,56**
trnN-GUU	0,00	0,00	0,00	0,00
Intergene20	*3,02*	*5,42*	*3,27*	*4,03*

The bolded rows marked intergenic spacers there LRT for the branch site model A was significantly better than the model B. (Model A was used for the ribosomal RNA and non-coding regions and supposes that substitutions in a DNA sequences of different tree branches are homogenous, model B – the same but supposes non-homogenous substitutions, see also materials and methods)

An unusual 1663 bp insertion was found *rrn16* and *rps12* genes of *Woodwardia unigemmata* (coordinates 93,404...95067). *W. unigemmata* is a fern of family *Blechnaceae* (Eupolypods II), whose plastome was sequenced by Lu et al., 2015 [41], and no genes were annotated in this region previously. The large part of *W. unigemmata* insertion has high sequence similarity to the insertion of *Lepisorus clathratus* (Polypodiaceae, Eupolipods I) located in the same region of IR, between *rrn16* and *rps12* genes. About 1160 bp of *W. unigemmata* and *L. clathratus* insertions demonstrated 65–78% similarity to each other. No similar sequences were found in IR of other Polypodiales, except for small (about 150 bp) sequence in *Matteuccia struthiopteris* (Onocleaceae, Eupolypods II) which has 62–66% similarity to *L. clathratus* and *W. unigemmata* insertions (further called WL-sequences).

**Fig. 4:**
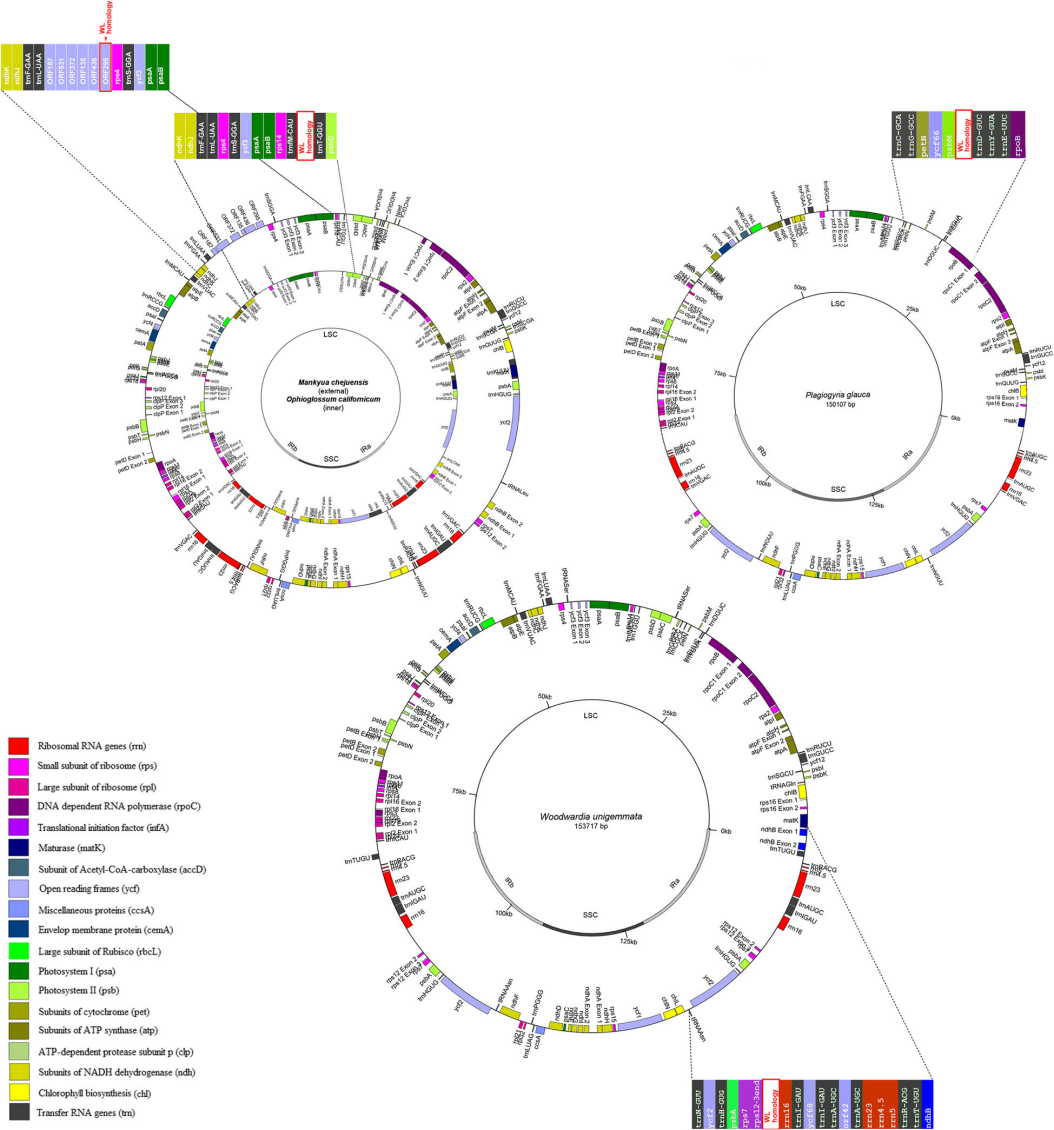
Location of WL-homologous fragments in plastomes of Ophioglossales*,* Cyatheales and Polypodiales ferns

Surprisingly the WL-like sequences were found in LSC regions of species from distant taxonomic groups of ferns, outside Polypodiales (Table 2). Firstly, small part of WL sequence has similarity to the fragment of LSC plastome of *Plagiogyria* species (*Plagiogyria* is a single genus in monotypic family Plagiogyriaceae, Cyatheales, see for example «Flora of China» [42]). To be more precise, the 772 bp part of WL sequence has 67% identity to the region of the *P. glauca* plastome (coordinates 29,128...29895, KP136831) and to *P. japonica* plastome (partial sequence, coordinates 4503–5273, HQ658099). In both species sequences with homology to WL lie within *trnD-GUC*-*psbM* intergenic spacer. Other ferns that also contain WL-like sequences in LSC are *Ophioglossum californicum* (KC117178) [34] and *Mankyua chejuensis* (KP205433). Both these species belong to Ophioglossales (basal ferns); they are distant from Polypodiales (core leptosporangiate ferns) and from Cyatheales. The fragments with similarity to WL sequences in Ophioglossales ferns have about 255–275 bp length and are located in the different regions of LSC. In *O. californicum* (KC117178) it is found in intergenic spacer between the *trnT-GGU* and *trnfM-CAU* genes, in *M. chejuensis* (KP205433, JF343520) it is also located in LSC region but in the other intergenic spacer (between *trnL-UAA* and *rps4*). It is annotated as *ORF295* (Fig. 4). Altogether, this suggests that there is a translocation of DNA fragments from LSC to IR (or vice versa) during evolution of fern plastomes.

**Table 2:** Coordinates of insertions with size 45 bp more inside Polypodiales IRs

№	Clades	Species	Insertion IR coordinates		Insertion size	Location	Genbank accession number	Description
			start	end				
1	1Eu*	*Dryopteris blanfordii*	7615	10,000	2385	Large insertion between *trnI-GAU* and *rrn16*	This work	Contains duplicated rrn16 and trnF-GAA genes
2	1Eu	*Polypodium glycyrrhiza*	9546	11,764	2218	intergene14 **(** *rrn16*-*rps12*)	KP136832	~ 700 bp part of insertion has homology to insertion of *L. clathratus*
3	1Eu	*Lepisorus clathratus*	10,863	14,073	3210	intergene14 (*rrn16*-*rps12*)	KY419704	Part if insertion has homology to part of WL and another to insertion of *P. glycyrrhiza*
4	1Eu	*Cyrtomium devexiscapulae*	22,596	22,968	372	intergene19 (*ycf2*-*trnN-GUU*)	KT599100	No significant homology to known sequences was found
5	1Eu	*Cyrtomium falcatum*	22,570	22,942	372	intergene19 (*ycf2*-*trnN-GUU*)	KP189363	No significant homology to known sequences was found
6	2Eu	*Cystopteris protrusa*	6100	6286	186	*rrn23*	KP136830	Duplication inside IR (homologous to 6403–6560)
7	2Eu	*Cystopteris protrusa*	6897	9498	2631	*rrn23*	KP136830	~ 950 bp fragment of insertion has 75–76% homology to mitochondrion of different mosses
8	2Eu	*Asplenium pekinense*	10,447	12,688	2316	intergene14 (*rrn16*-*rps12*)	KY427331	~ 200 bp of insertion has homology to *rrn16*-*rps12* spacer of *L. clathratus*
9	2Eu	*Rhachidosorus consimilis*	10,780	11,683	903	intergene14 (*rrn16*-*rps12*)	KY427356	No significant homology to known sequences was found
10	2Eu	*Woodwardia unigemmata*	11,015	12,678	1663	intergene14 (rrn16-rps12)	KT599101	WL sequence
11	2Eu	*Matteuccia struthiopteris*	13,885	14,255	370	intergene16 (*rps7*-*psbA*)	KY427353	Part if insertion has homology to part of WL sequence
12	2Eu	*Asplenium prolongatum*	22,083	23,414	1331	intergene19 (*ycf2*-*trnN-GUU*)	KY427332	The most of insertion has homology to part of *rrn16*-*rps12* spacer of *Polypodium glycyrrhiza*
13	Pt	*Ceratopteris richardii*	14,185	14,499	314	intergene18 (*trnH*-*ycf2*)	KM052729	No significant homology to known sequences was found
14	Pt	*Cheilanthes lindheimeri*	22,714	25,096	2382	intergene19 (*ycf2*-*trnN-GUU*)	HM778032	No significant homology to known sequences was found

^*^ *1Eu* Eupolypods I, *2Eu* Eupolypods II, *Pt* Pteridaceae

Interestingly, the part of the WL sequence has high identity (67%) to the 203-bp fragment of about 5,9 Kb intergenic spacer between *tRNA-CGA* and *tRNA-TTT* genes of mitochondrial genome of *Asplenium nidus* (partial sequence, coordinates 3188…2986, AM600641) (Table 2). *Asplenium* is a genus that belongs to the same clade of Polypodiales - eupolypods II as *Woodwardia* but to the other family - Aspleniaceae. We performed similarity search of WL-sequence against fern mitochondrial contigs available in Utah State University Repository [45; http://digitalcommons.usu.edu/fern_genome/]. Two hits were found in *Plagiogyria formosana* - 244 bp (identity 72%, contig №439, coordinates 5336–5585) and 277 bp (identity 72%, contig №93, coordinates 1–277). No hits were detected in the contigs of another available fern species (*D. conjugata*, *Pteridium aquilinum*, *C. richardii*, *Polypodium glycyrrhiza, C. protrusa*).

We have estimated relative evolution rate of genes and intergenic spacers of Polypodiales IR. For protein-coding genes we analyzed dN/dS using branch-site model, for non-coding regions two models: homogenous substitution parameters and non-homogenous were used (see materials and methods for details). We found that for four non-coding (intergenic) regions the model, which has different substitution parameters for different branches, had significantly higher likelihood (LRT) compared to model based on assumption of identical substitution parameters in different lineages (see Table 3). All these regions are spacers: between *trnA-UGC* and *or*f42 (intergene8), between *rrn16* and *rps12* (intergene14)*,* between *rps7* and *psbA* (intergene16), and the last one between *ycf2* and *trnN-GUU* (intergene19).

**Table 3:** Coordinates, size and identity of WL-homologous regions in different species

Species	GenBank accession number	Region	Coordinates	Annotation	% Identity to WI-homologous fragment of *W. unigemmata*	b.p.
*Plagiogyria glauca* plastid, partial	KP136831	LSC	29,128...29895	intergenic spacer	67%	768
*Plagiogyria japonica* plastid, partial	HQ658099	LSC	4503…5273	intergenic spacer	67%	770
*Mankyua chejuensis* plastid, complete genome	KP205433	LSC	62,408...62158	ORF295	66%	255
*Ophioglossum californicum* plastid, complete genome	KC117178	LSC	49,930...49660	intergenic spacer	65%	270
*Asplenium nidus* mitochondrion, partial	AM600641	Mitochondrion	3188…2986	intergenic spacer	67%	202

## Discussion

In contrast to previous observations on the stability of the IR region, we found high variability in IR sequence and gene content in Polypodiales ferns. There are two hypervariable regions – one located at the beginning of IR, 0–3 Kb, and the second is 7–11 Kb region. These regions are the subject to the similar evolutionary changes occurred independently in the different clades. The first region in most species contains tRNA pseudogene. The members of both Eupolypods I and Eupolypods II demonstrated independent deletion of *trnI-GAU*-*ycf68* region (i.e. *Dryopteris* and *Onoclea*). Polypodiales (together with Salviniales and Cyatheales) belong to a clade called core leptosporangiates [43]. Their plastomes acutely differ from those of eusporangiate ferns (Psilotales or whisk ferns, Ophioglossales, Marattiales, Equisetales) [8, 33]. It should be noted however that comparative analysis of fern plastomes is obfuscated by the uncertainty of the annotation of tRNA genes. This concerns, in particular, the intron-containing *trnT-UGU*, which was reported in IR (between *ndhB* and *trnR-ACG*) of several fern plastomes [37, 43, 44] and thought to be specific feature of core leptosporangiates. But intron-containing *trnT-UGU* was not found in other fern lineages [45, 46] or in any other plants outside ferns; only intronless *trnT-UGU* is present*.* This is unusual given that plastid tRNA genes, in contrast to protein-coding genes, have highly conserved exon-intron structure. Gao and coworkers supposed that tRNA genes may be lost repeatedly independently during evolution of ferns and probably the loss of *trnT-UGU* is the one of those events [45]. Our analysis which included manual reannotation and check using tRNA prediction program tRNAscan-SE however does not support the functionality of intron-containing IR-located *trnT-UGU* in any Polypodiales species where it was reported. In contrast, we found the *trnT-UGU* pseudogene in the IR of almost all Polypodiales. Most likely, this pseudogene is difficult to be recognized and therefore results of automatic annotation could be interpreted as gene loss. Notably, Gao and co-workers [45] compared the sequences of putative intron-containing and intronless *trnT-UGU* and it can be seen that the former are unusually divergent, much higher than expected for a functional tRNA gene. We conclude that intron-containing IR-located *trnT-UGU* is an artifact caused by the shortcomings of the automatic annotation. Moreover, two parts (“exons”) of this pseudogenes can be recognized by automation annotation programs, such as DOGMA, as different tRNAs - *trnT-UGU* and *trnL-CAA*.

In the second hypervariable region, 7–11 Kb, we found an unusual insertion (the WL-sequence) in two unrelated Polypodiales – *Woodwartia unigemmata* and *Lepisorus clathrathus*. Smaller insertion with high similarity to the WL-sequence was found in the same region in *Mattheucia sthruttiopteris*. In addition, the insertions with high similarity to WL-sequence were found in plastomes of Ophioglossales (basal ferns, distant from Polypodiales) but in different position – in LSC region.

The WL-sequence has high similarity with the region of mitochondrial genome of *Asplenium nidus*. This has two possible explanations: that it is either the sequence of mitochondrial origin, which was integrated in the plastome, or the sequence of plastid origin, which was integrated into mitochondrial genome and lost from the plastid genomes of most ferns, with exception of *W. unigemmata* and *L. clathrathus*. By now we can’t make a conclusion about which of these two hypotheses is true, due to the unavailability of fern complete mitochondrial genome sequences.

In any case, it is a result of the horizontal genome fragment transfer between mitochondria and plastids. Horizontal genome fragment transfer is a phenomenon, commonly observed in the pro- and eukaryotes. In plants, the presence of three genomes within a cell compartments (mitochondria, chloroplast and nucleus) leads to different possible types of intracellular genome fragments exchange: between organelles and nucleus and between mitochondria and chloroplasts, bidirectional [47]. The transfer of genetic material from organelles to the nucleus seems to be a continuing evolutionary process of the prokaryotic ancestors’ genome reduction [48, 49]. Many reports asserted that plant mitochondrial genomes are unusually prone to the introgression of alien sequences compared to chloroplast and nuclear genomes [47, 50]. There are only few data on mitochondria of ferns. No complete mitochondrial genome assemblies are available, only contigs. Multiple regions with strong sequence similarity to plastid DNA were detected by [51] but they didn’t relate to the plastome sequences in the total genomic contigs of six ferns species *Dipteris conjugata* (Gleicheniales), *Plagiogyria formosana* (Cyatheales), *Pteridium aquilinum* (Dennstaedtiaceae), *Ceratopteris richardii* (Pteridaceae), *Polypodium glycyrrhiza* (eupolypods) and *Cystopteris protrusa* (eupolypods). Authors speculated that the plastome-like sequences reside within the nuclear or mitochondrial genomes [42]. Assuming this is the case, it implies that the horizontal transfer of organelle genome fragments are not rare events in the evolution of ferns.

## Conclusions

In this study we investigated the structure and evolutionary stability of IRs of plastomes in Polypodiales ferns. The two regions of IRs were found to be highly variable: (i) the sequences between *ndhB* and *trnR-ACG* genes (~3 Kbp) and (ii) the fragment including the *rrn16* gene and flanking vicinity regions (~4,5 Kbp). This blinking of *trnI-CAU*, *trnT-UGU*, *ndhB* and *rps12, trnI-GAU, ycf68, rrn16* genes related to these regions was observed in different species. The plastomes of three *Dryopteris* species demonstrate dynamic process of trnI-GAU elimination/*rrn16* duplication.

Two Polypodiales species - *W. unigemmata* and *L. clathratus* - have an unusual sequence in the IR region. It demonstrates similarity to LSC spacers *trnL-rps4* of Ophioglossales and *pbsM-trnD* of Cyatheales and with the part of mitochondrial genome of *Asplenium* (Polypodiales). We suppose these features are a consequence of intraplastomic rearrangements as well as of the transfer between the chloroplast and mitochondrial genomes during the evolution of ferns.

## Methods

### Plant material

Mature fronds of both *Dryopteris filix-mas (L.) Schott*, *Dryopteris blanfordii* (C. Hope) C. Christensen and *Dryopteris villarii (Bellardi)* Woyn. ex Schinz & Thell were sampled from outdoor section of the Moscow State University Botanical Garden.

*Dryopteris filix-mas* (L.) Schott is a common fern species in the Russian forests, therefore the specimen’s collection locality was stated only approximately as “in the vicinity of Moscow”.

*Dryopteris blanfordii* (C. Hope) C. Christensen grows in *Picea* or *Abies* forests at 2900–3500 m AMSL in China (Gansu, Sichuan, Xizang, Yunnan), Afghanistan, India, Kashmir, Nepal, and Pakistan [52–54]. The parent plant was collected in 2003 in India. Spores of the specimen were germinated under artificial conditions of the greenhouse of Botanical Garden of the Moscow State University. Developed sporophytes were then transplanted to the outdoor section of the Botanical Garden.

*Dryopteris villarii (Bellardi)* Woyn. ex Schinz & Thell*.* - subalpine species, grows on outcrops of hills, limestone cliffs, including high-mountain in Central and SouthEurope [55]. The spores, courtesy of Zürich Botanical Garden seed department (collected in natural habitat of Swiss Confederation), was germinated and specimen was germinated and grown in small greenhouse of Moscow State University Botanical Garden during 2013–2017.

*Adiantum hispidulum* Sw. pantropical, paleotropical species, it is distributed from eastern Africa through southern India, Thailand and the Ceylon to Pacific islands, Polynesia, New Zealand and Australasia [56–59]. The adult frond of *Adiantum hispidulum* was collected from greenhouse of Botanical Garden of Moscow State University. The voucher specimen was kept in Herbarium of Biology Department of Moscow State University.

### Chloroplast genome sequencing, de novo assembly and annotation

The chloroplast DNA (cpDNA) were sequenced using the Illumina MiSeq high-throughput sequencing platform. For a sample preparation, the adult live plants were taken from the collection of the Moscow State University Botanical Garden. cpDNA was extracted from 2,6 g. (fresh weight) of fronds using the cpDNA extraction protocol [60, 61] with small modifications: after cleaning with a distilled water, the fronds were homogenized in 35 ml isolation buffer at +4 °C (Tris-HCl (pH 8,0) 50 mM, EDTA 7 mM, 1% PVP-40, NaCl 1,25 M, ascorbic acid 0,25 M, sodium metabisulfite 10 mM, Borax 0,0124 M) and the homogenate was filtered using soft wipes. The homogenate was then successively centrifuged at 200 g for 15 min at 4 °C (cell wall debris was discarded), at 1000 g for 20 min at +4 °C (the precipitate was discarded) and finally at 2000 g for 20 min at +4 °C. In the latter case, the precipitate was resuspended in 3 ml of wash buffer (Tris-HCl (pH 8,0) 50 mM, EDTA 25 mM) and carefully loaded into a 15 ml tube containing sucrose gradient consisting of 7 ml of 52% sucrose in wash buffer and overlaid 4 ml of 52% sucrose in wash buffer. The tube with the sample and sucrose gradient was centrifuged at 3500 g for 60 min at 4 °C. The interface between 52% and 30% sucrose (about 1 ml) was collected, centrifuged at 12,000 g. The pellet was resuspended in 900 μl of wash buffer and 100 μl of 10% CTAB was added for lysis (1 h, 55 °C). Then the DNA purification step was carried out using the protocol described in [62].

The TruSeq protocol (NEBNext® DNA Library Prep Master Mix Set for Illumina, E6040, NEB reagents) was used for preparing the genomic libraries. We made PE sequence (2 × 300 bp.) with a double number of each library reads about 1.2–1.97 M. After the quality trimming with Trimmomatic [63], sequencing reads were filtered using 13 complete and 5 partial fern chloroplast genome sequences from RefSeq database and Bowtie2 [64]. Then the two contig sets were produced for both filtered and unfiltered reads sets using the Velvet Assembler [65] and MIRA4 [66]. Assembled contigs and scaffolds were selected for the next assembly if they showed similarity to the published fern chloroplast genomes. The final de novo assembly was finished through a few iterative steps. The draft sequence was manually corrected by the PE reads mapping.

We have obtained the reads of complete circulated chloroplast genomes comprising the large single-copy region (LSC), small single-copy region (SSC) and the two inverted repeat (IR) regions. Finally, mapping of the initial reads was performed to the assembly in order to check for the potential assembly artefacts. Protein-coding gene annotation in the assembled chloroplast genome was annotated by DOGMA [67]. Bowtie2, VarScan (v.2.3.7) and SAMtools/BCFtools software packages were used for mapping of the reads and variant calling [64, 68, 69].

### Chloroplast genomes analysis

Genbank or ENA accession numbers of sequences included in this study are listed in Table 2.

Analysis of the complete chloroplast genomes was carried out on species sequenced in this study together with previously reported species. Nine plastomes were downloaded from the GenBank. A complete list of the analyzed species can be found in the Table 1. Firstly, sequences of all the chloroplast sequences were pair-wise aligned against each other by Kalign (www.ebi.ac.uk/Tools/msa/kalign). Phylogenetic analysis was carried out by a maximum likelihood (ML) using Mega 6.0 [70]. Comparative analysis of chloroplast genome sequences was performed by the mVista web-tool (http://genome.lbl.gov/vista/mvista/submit.shtml).

For evolution rate analysis for the each region of IRs (genes and intergenic regions separately), as well as for the concatenate of all coding sequences, the alignments was built using MUSCLE [71]. An ML tree was constructed using concatenate alignment. Substitution model with lowest BIC score was chosen using modelTest function from phangorn package [72]. The tree topology was optimized using follow parameters: the nucleotide substitution matrix, gamma, the proportion of invariant sites and gamma distribution parameter. For non-protein coding regions the tree branch lengths were calculated by two models (homogenous substitution parameters - nhomo = 1, non-homogenous - nhomo = 4) using baseml [73], then models were compared by LRT. For protein-coding regions, dN and dS were estimated for each gene using codeml from PAML package [73], dN/dS ratio in each lineage was estimated by branch and M0 model. For both baseml and codeml analysis phagorn concatenate tree with nearest neighbour interchange was used. Distance matrices were calculated using baseml/codeml trees in the ape package [74]. Then relative evolution rate for each region (coding and non-coding) was calculated using ERaBLE [75]. Sliding window analysis (window = 200 b.p.) of p-distances, i.e. the proportion of nucleotide differences per site between sequences was calculated by perl script made by Masafumi Nozawa [76].

## Additional file

Additional file 1: List of genes annotated for *D. filix-mas* and *D. blanfordii* chloroplast genome by DOGMA. (XLSX 46 kb)
